# Early results of a novel modular knee arthrodesis implant after uncontrolled periprosthetic knee joint infection

**DOI:** 10.1186/s12891-023-07016-2

**Published:** 2023-11-15

**Authors:** Kadir Büyükdoğan, Yusuf Öztürkmen, Barlas Goker, Melih Oral, Tolga Atay, Korhan Özkan, Ömür Çağlar, Mehmet Ayvaz

**Affiliations:** 1 Department of Orthopedic Surgery, Güven Hospital, Şimşek Sokak, No: 29, A.Ayrancı, Ankara, Turkey; 2 Department of Orthopedics and Traumatology, Istanbul Training and Research Hospital, Istanbul, Turkey; 3 Department of Orthopedics and Traumatology, Faculty of Medicine, Hacettepe University, Ankara, Turkey; 4 Department of Orthopedics and Traumatology, Faculty of Medicine, Suleyman Demirel University, Isparta, Turkey; 5 Orthopedics and Traumatology Department, Faculty of Medicine, Medeniyet University, Istanbul, Turkey

**Keywords:** Knee Arthrodesis, Total knee arthroplasty, Periprosthetic joint infection, Modular intramedullary nail

## Abstract

**Aim:**

The aim of this study is to evaluate the functional outcomes and complications after non-fusion knee arthrodesis with a modular segmental intramedullary implant used for infected total knee arthroplasty revisions.

**Methods:**

A retrospective review of the patients who had been surgically treated with a modular intramedullary arthrodesis implant for recurrent infection after revision TKA between January 2016 and February 2020 were included. The indications for arthrodesis were failed infected TKA with massive bone loss, deficient extensor mechanism and poor soft tissue coverage that precluded joint reconstruction with revision TKA implants. Clinical outcomes were assesed with visual analogue scale for pain (pVAS), Oxford knee score (OKS) and 12-item short form survey (SF-12). Full-length radiographs were used to verify limb length discrepancies (LLD).

**Results:**

Fourteen patients (4 male and 10 female) patients with a mean age of 69.3 (range, 59 to 81) years at time of surgery were available for final follow-up at a mean of 28.8 months (range, 24–35 months). All clinical outcome scores improved at the final follow-up (pVAS, 8.5 to 2.6, *p* = .01; OKS, 12.6 to 33.8, *p* = .02; SF-12 physical, 22.9 to 32.1, *p* = .01 and SF-12 mental, 27.7 to 40.2, *p* = .01). The mean LLD was 1.0 cm (range, + 15 – 2.3 cm). Re-infection was detected in three patients (21.4%). Two patients were managed with suppressive antibiotic treatment and a third patient required repeat 2-stage revision procedure. In one patient, a periprosthetic femur fracture was observed and treated with plate osteosynthesis.

**Conclusion:**

Uncontrolled infection after total knee arthroplasty can be effectively treated with arthrodesis using a modular intramedullary nail and satisfactory functional results can be obtained.

**Level of evidence:**

Level 4, Retrospective cohort study.

## Introduction

Infection is a devastating complication of total knee arthroplasty (TKA) with reported frequencies of up to 3% in large patient series [1]. In high-risk patients with additional risk factors (rheumatoid arthritis, diabetes, long-term antibiotic use, etc.) the likelihood of infection can go up to 5–15% [2]. The management of infected TKA management often presents a challenge to surgeons. Revision TKA may not always be viable for the patient, mainly due to failure of eradication of the infection [3]. In such cases, knee arthrodesis provides an alternative method of limb salvage.

Arthrodesis for infected knee arthroplasty can provide a stable and painless limb. The indications for knee arthrodesis are limb salvage after failed TKA revision surgery, deficient extensor mechanism and high functional demand in young patients, poor soft tissue coverage, and severe infections with highly virulent microorganisms that preclude joint reconstruction with arthroplasty implants [4]. Several techniques for knee arthrodesis have been described: external fixation, intramedullary nailing, plate fixation, and modular intramedullary implants [5–8]. Modular segmental implants allow immediate weight bearing without the need of bony fusion, and minimize leg length discrepancy (LLD). Some designs also provide reconstruction for the metaphyseal bone loss by bridging the large gaps between the femurs and tibiae. The aim of this study is to evaluate the functional outcomes and complications after non-fusion knee arthrodesis with a modular segmental intramedullary implant used for infected TKA revisions.

## Materials and method

Hacettepe University Ethical committee approved the study and waived the requirement to obtain informed consent (Protocol no: 2020/13–53, Date: 08/25/2020). This study was conducted in line with the principles of the Declaration of Helsinki. A retrospective review of the medical records was performed at five different centers between January 2016 and February 2020. Patients who had been surgically treated with a modular segmental intramedullary arthrodesis implant (ESTAS, Sivas, Turkey) for recurrent infection after revision TKA were included. Indications for arthrodesis failed infected TKA with massive bone loss, deficient extensor mechanism and poor soft tissue coverage that precluded joint reconstruction with revision TKA implants. A staged arthrodesis was performed in all patients. No patients were excluded because of previous operations, comorbidities or previous infection. The demographics, comorbidities, follow-up duration, previous treatments, and resection lengths were obtained from patient charts. Functional status of the patients at the final follow-up, and complications encountered within follow-up were recorded. The Charlson comorbidity index was calculated for each patient [9].

The diagnosis of PJI was based on previously published criteria set by International Consensus Meeting on PJI (2013) [10, 11]. All patients fulfilled the criteria of Musculoskeletal Infection Society (MSIS) for PJI. After confirming PJI, a staged revision was performed for all patients. This included removal of the all implants followed by an extensive debridement. After obtaining at least five tissue specimens in areas showing signs of infection, empiric intravenous antibiotic treatment was initiated intraoperatively. Antibiotic impregnated polymethylmethacrylate (PMMA) (2 gr vancomycin per cement pack) with two titanium rods was used as both spacer and temporary arthrodesis. Intravenous antibiotic treatment was administered for 6 weeks and was tailored according to intraoperatively detected pathogen and its susceptibility pattern. Patients were followed up for any signs of ongoing infection using clinical assessment and inflammatory markers including C-reactive protein, sedimentation rate and leukocyte count in blood. Repeat debridement and spacer exchanges were performed in the cases of ongoing infections. No joint aspiration performed prior to arthrodesis. When there was no clinical sign of infection, decisive knee arthrodesis with a modular system was performed.

### Operative technique

**Fig. 1:**
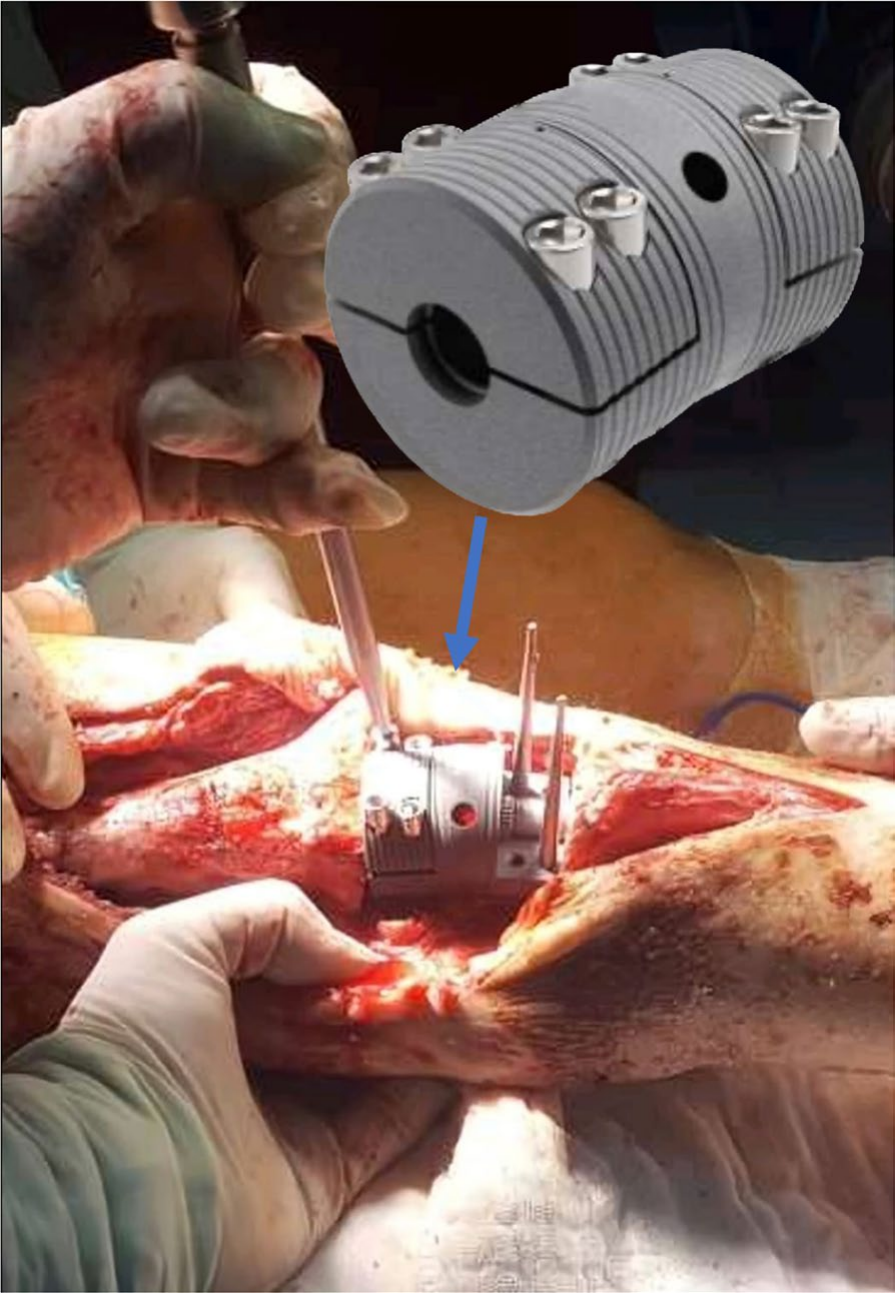
Implant design consists of two separate intramedullary nails at the edges with a spacer in between. The spacers are semi-circular shells that are connected with 5 mm locking screws

All patients treated with a modular segmental intramedullary arthrodesis implant (ESTAS, Sivas, Turkey). This implant upgrades on previous designs by an improved clamp mechanism. Implant is CE certificated (M.2021.106.14229) and all components of the system are manufactured from Ti6Al4V ELI alloy (ASTM F136, ISO 5832–3). The implant design consists of two separate intramedullary nails at the edges with a spacer in between (Fig. 1). The spacers are semi-circular shells that are connected with 5 mm locking screws. Additionally, there are screw holes at the end of stems to allow locking screw insertion to increase rotational stability. Surface treatment applied to stems include grit blasting with abrasive aluminum-oxide (Al2O3) sands to increase the surface area and passivating the surface by sequential anodization to form a controlled TiO2 layer on stems. This process increases the physical hydrophilicity of the surface and achieves the roughness that allows appositional bone growth for secondary stability.

After reaming femoral and tibial medullary canals, appropriately sized stems were press-fit implanted to ensure primary stability. According to surgeon’s preferences, locking screws were used to ensure rotational stability. The contralateral limb length was the target length of the operated knee, however soft tissue tension that allows uneventful wound closure was the primary indication to set the length of the modular system. Bone contact was not targeted and no attempt was made to fill the space between the femur and tibia.

After surgery, patients were mobilized with a help of crutch or walker. At early post-operative period patients were encouraged to weight bear as tolerated. Loading is gradually increased and full weight bearing was reached after 4 weeks.

### Outcome evaluation

**Fig. 2:**
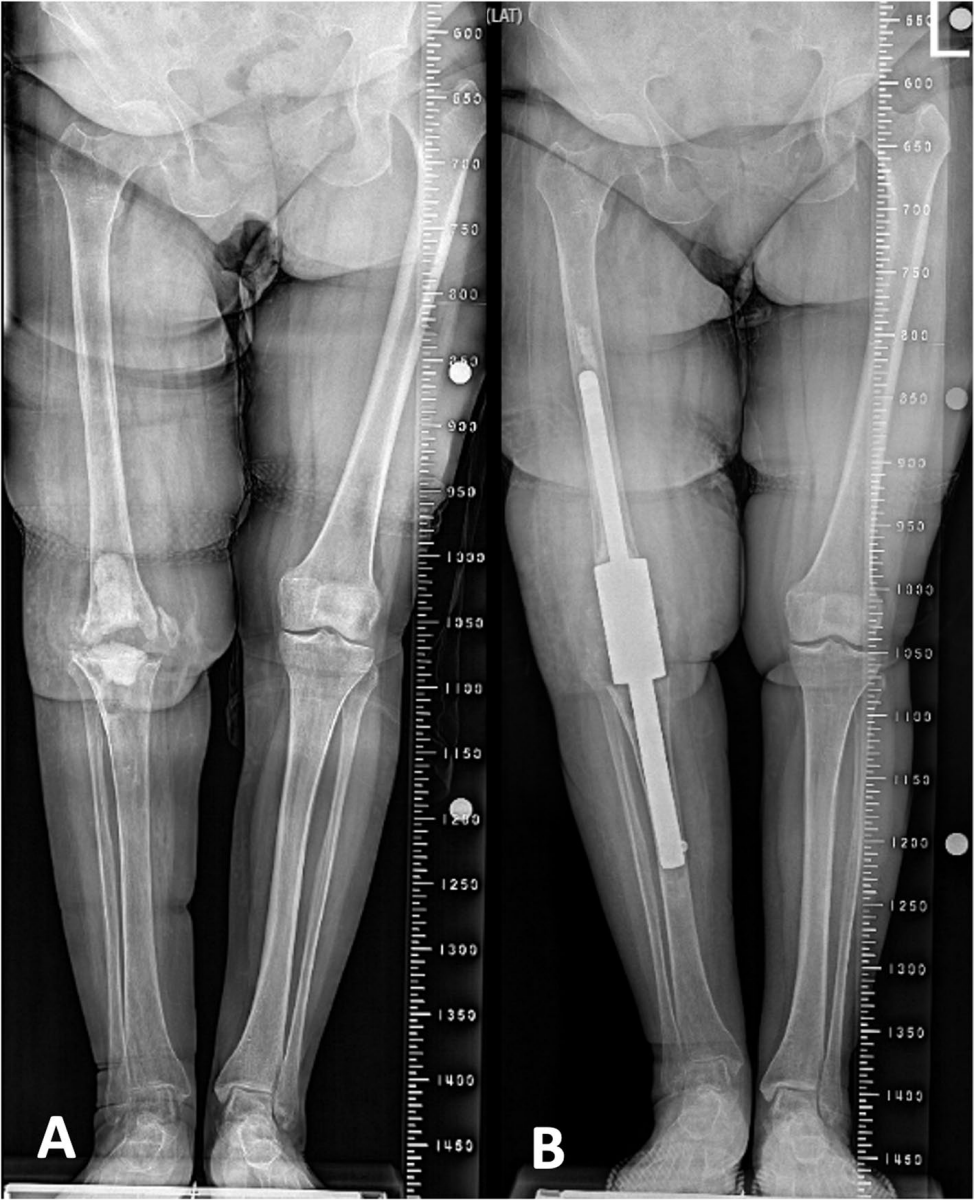
Pre-operative (**a**) and post-operative (**b**) full-length radiographs of a patient who underwent knee arthrodesis with a modular knee arthrodesis sytem after uncontrolled periprosthetic knee infection

Patients were evaluated at their final follow-up by a physician who joined their treatment. Pain assessment was done by the visual analogue scale (VAS). Functional evaluation of the patients was performed using the Oxford Knee Score (OKS). OKS is a patient-reported outcome measure of that evaluates the pain and physical capabilities in patients who underwent TKA [12]. Turkish version of this questionnaire has been found to be reliable and valid [13]. Physical and mental well-being was determined by the 12-Item Short Form Health Survey (SF-12). Bilateral full-length radiographs were used to verify LLD (Fig. 2).

A full recovery was defined as presence of all the following: normal ESR and/or CRP, no evidence of inflammation of the scar and no sinus tract, no further antibiotic therapy, and no revision surgery [14]. Bone union was not among our objectives, given the severe bone loss in some of our patients.

SPSS® Statistics 23 (IBM Corp., Armonk, New York, USA) was used for statistical analysis. Categorical variables were presented as numbers and percentages while continuous variables were given as mean and range for descriptive statistics. Wilcoxon’s rank sum test for matched pairs were used to compare pre-operative and post-operative outcome measurements, because data was not normally distributed. The effect sizes of the Wilcoxon’s rank sum tests were calculated as absolute *r* = Z/ √n. Absolute r values and strengths of correlation were interpreted as weak (*r* = 0.1), medium (*r* = 0.3), or large (*r* = 0.5) according to the Cohen classification. The level of significance was set at 0.05, and all *p* values were two-tailed.

## Results

Four male (29%) and 10 female (71%) patients with a mean age of 69.3 (range, 59 to 81) years at time of surgery met our inclusion criteria and were available for final follow-up at a mean of 28.8 months (range, 24–35 months) (Table 1). The most common presenting symptom was pain (100%) in all patients. The mean length of resection was 77.1 mm (range, 50 to 130 mm). The mean proximal stem length was 14 cm and the mean distal stem length was 17 cm.

**Table 1:** Demographics of the patients

Case	age	sex	Previous operations (n)	Infecting agent	Indication for arthrodesis	Charlson index	F/U (months)	LLD (mm)	Complications
1	73	F	2	MRSA	Massive bone loss	4	29	-15	
2	81	M	3	S. *Aureus*	Massive bone loss	7	32	-10	
3	68	F	3 (including failed arthrodesis with plate fixation)	P.*Aeruginosa*, E. *Faecalis*	Extensor mechanism deficiency	6	24	-18	Re-infection, suppressive antibiotic treatment
4	69	F	4	P.*Aeruginosa,* S. *Aureus*	Massive bone loss & extensor mechanism deficiency	4	33	-16	
5	65	M	2	S. *Aureus,* C*. albicans*	Massive bone loss	3	29	+ 15	
6	71	M	2	MRSA	Massive bone loss	5	30	-20	Re-infection, suppressive antibiotic treatment
7	60	F	1	MRSA	Massive bone loss	4	26	-23	
8	77	F	3	S. *Aureus*	Soft tissue defect	4	32	+ 15	
9	66	F	3 (including failed external fixation osteosynthesis)	S. *Aureus*	Extensor mechanism deficiency	5	30	-20	Repeat 2-stage revision
10	73	F	2	S.*Marcescens*, E. *Faecalis*	Massive bone loss	7	25	-13	Periprosthetic femoral fracture- plate osteosynthesis
11	59	F	2	CNSA	Massive bone loss	4	24	-12	
12	67	F	3	S. *Aureus*, Klebsiella	Soft tissue defect	4	30	+ 5	
13	74	F	3	E.*Coli*, S. *Aureus*	Extensor mechanism deficiency	4	25	-16	
14	68	M	4	CNSA, C.*Albicans,* P.*Aeruginosa*	Massive bone loss & extensor mechanism deficiency	4	35	-18	

*MRSA* methicillin-resistant Staphylococcus aureus, *CNS* coagulase-negative Staphylococcus

The mean LLD was 1.0 cm (range, + 1.5 – 2.3 cm). All of patients were able to bear full weight, and 11 (78%) had no pain during ambulation. 4 patients were able to ambulate independently without the assist of canes or crutches. 6 patients had to use canes for ambulation, and four required crutches.

The mean OKS score increased from the preoperative value of 12.6 to 33.8 (*p* = 0.02, effect size 0.87). The mean VAS score was 8.5 preoperatively, which decreased to 2.6 at final evaluation (*p* = 0.01, effect size 0.87). The mean SF-12 physical score increased from the preoperative value of 22.9 to 32.1 (*p* = 0.01, effect size 0.78). The mean SF-12 mental score improved from 27.7 to 40.2 at the final follow-up (*p* = 0.01, effect size 0.87) (Table 2).

**Table 2:** Outcome scores

	SF-12 Physical	SF-12 Mental	pVAS	OKS
Pre-op	22.9 ± 1.3	27.7 ± 4.2	8.5 ± 1.0	12.6 ± 4.8
Post-op	32.1 ± 8.1	40.2 ± 4.3	2.6 ± 0.5	33.8 ± 6.5
*p* value (effect size)	.01 (0.78)	.01 (0.87)	.01 (0.87)	.02 (0.87)

*SF-12* 12-item Short Form Survey, *pVAS* Visual Analog Score for pain, *OKS* Oxford Knee Score

**Fig. 3:**
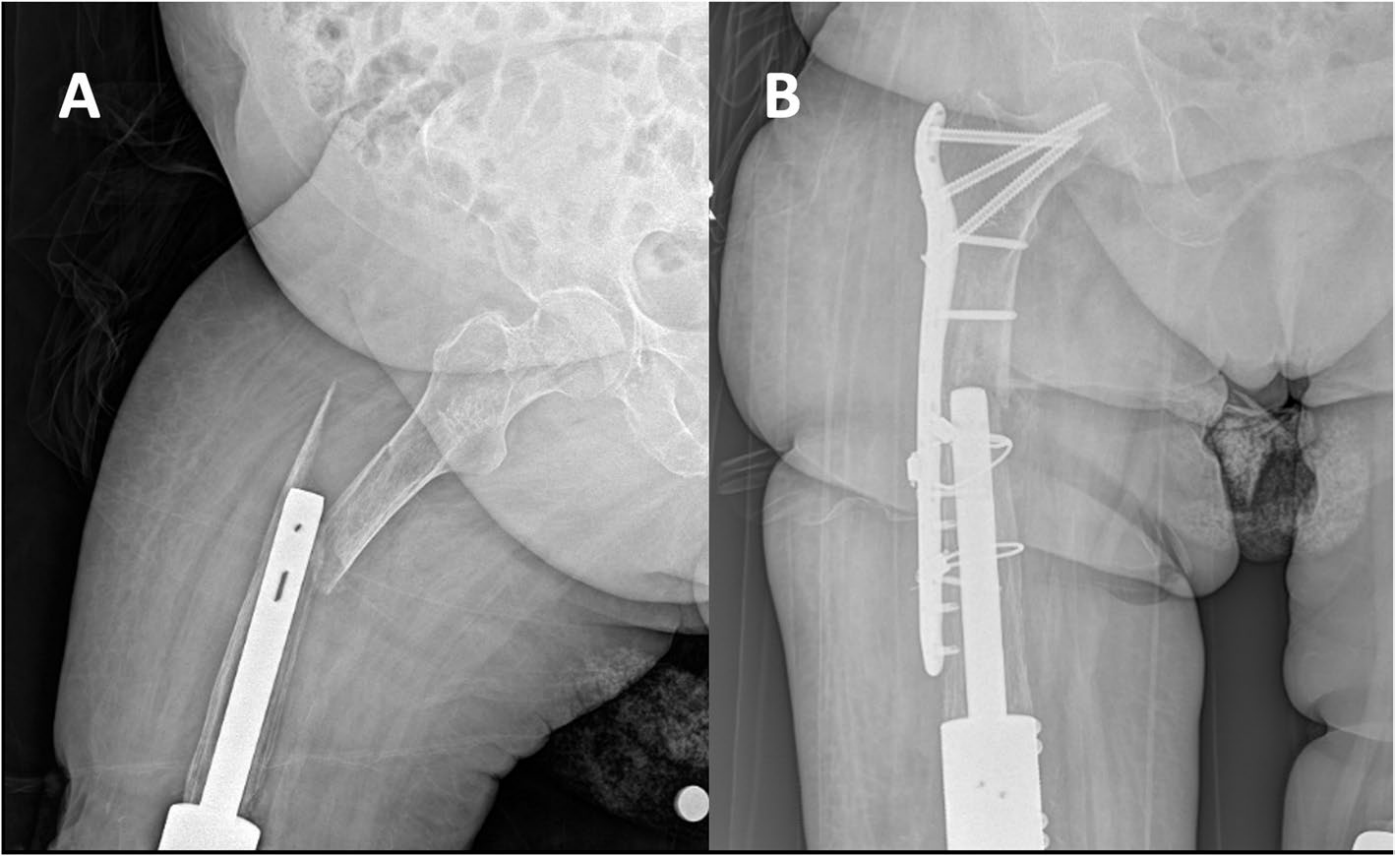
**a** Radiographs depicting a periprosthetic fracture in proximal femur after knee arthrodesis and (**b**) internal fixation of fracture with a plate

Re-infection was detected at three patients (21.4%). In two patients, one of whom had been using corticosteroids for the past decade due to temporal arteritis and another who was receiving hemodialysis for chronic kidney disease, chronic infections were managed with suppressive antibiotic treatment. In the third patient, who had a diagnosis of rheumatoid arthritis and previously underwent external fixation arthrodesis, re-infection was detected at 10 months after index procedure. Intramedullary nail removal was performed and a second step of repeat two-stage revision was scheduled at the time of manuscript preparation. In another patient, periprosthetic femoral fracture was observed after falling down from a chair 6 months after surgery. The patient was treated with open reduction and plate osteosynthesis, and had no signs of infection at the final follow-up (Fig. 3).

## Discussion

In this series, we evaluated 14 cases of modular knee arthrodesis for failed infected TKA management. The most important findings of this study are the observed improvements on the overall well-being, pain levels, functional status of the patients, and the low rate of reinfections and other complications. All of the patients in this cohort were able to bear full weight and mobilize independently, and four of them did not require assistive devices.

Nonunion is the most common complication of knee arthrodesis, which may affect up to a third of cases [15]. Although the occurrence is high, this can often be harmless; it has been hypothesized that the implant might work as an endoprosthesis in the case of nonunion [15]. In the case of modular intramedullary implants with extensive spacers, bony fusion is not always achieved and sought for [16]. Another benefit of the omission of bony fusion for larger spacers is avoidance of significant limb shortening, which is vital for the ambulation of patients who already have substantial deficits of bone stock. Modular segmental implants can also provide this by bridging massive bone losses. The implant used in this study shares a similar design with a modular intercalary endoprosthesis, which are differentiated by 10° of angulation for knee flexion in the arthrodesis system. The literature is scarce in terms of similar series. There are other modular knee arthrodesis systems similar to the study implant in terms of design such as the OsteoBridge™ IKA (Merete, GmbH, Berlin, Germany) (OIKA) and the Waldemar Link System (WLS) (Endo-Klinik Hamburg, Germany). These implants also have spacers that provide modularity and bridge the joint space for arthrodesis, and they have been shown to be successful for knee arthrodesis for various indications [17–19]. The implant used in the present study differs from these implants with an improved clamp mechanism. WLS uses two screws and locking bolts for the oblique plane engagement of the tapered clamps. In OIKA system, angled spacer is composed of upper and lower half-shells. Femoral and tibial nails are clamped within two parallel half- shells simultaneously.

The current system is composed of a fixed angled fully circular shell in the central part and two separate upper half-shells in the proximal and distal ends that ensures fixation of the arthrodesis (Fig. 1). Following the placement of tibial and femoral nails to central portion of the fixed part, the proximal and distal fixation is achieved by locking the proximal and distal half-shells sequentially. Although there are no biomechanical studies that investigate the current implant the clinical outcomes were non-inferior to the results of the aforementioned implants. Luyet et al. reported a postoperative VAS score a 2.8 in their cohort of OIKA-treated with a late-complication rate of 50% [20]. Additionally, Iacono et al. reported one intraoperative fracture in 22 patients with the insertion of the WLS nail [16]. Our series showed no intraoperative fractures, which may be explained by the design of the clamp mechanism, as it allows a third interface to fix the nails compared to the WLS nail. The omission of the cylindrical clamp mechanism in the design of OIKA theoretically reduces the risk of loosening or structural failure. In spite of this, currently there is not enough clinical evidence to illustrate this as it is not optimal to compare these implants based on the reported retrospective studies due to the relatively smaller numbers of cases.

Knee arthrodesis with large bone defects have been reconstructed with modular intercalary endoprostheses in a few studies [21, 22]. These reports have indicated early return to full weight bearing, painless mobilization, and unrestricted daily activities. The results of this study are consistent with the similar reports in the literature. Series of intramedullary knee arthrodesis without fusion have been mostly successful and comparable to those with bone fusion [5, 14, 16]. This study has a cohort which is most similar to that of Mayes et al. in terms of reconstruction [23]. Both studies stand out due to evaluating arthrodesis with large defect reconstruction without the aim of bone fusion; a manner which is similar to intercalary reconstructions.

Pain management is one of the most important aspects of infected TKA. All of the patients in this study presented with pain, and the mean VAS score was 8.5 before surgery. The implantation of the modular knee arthrodesis led to a 6-point drop in the average VAS score at the final follow-up assessment. Luyet et al. found a postoperative VAS score of 2.8 with a similar modular knee arthrodesis implant [20]. In another study with a modular intramedullary arthrodesis nail, the VAS scores were reduced from the preoperative mean of 7.9 to 2.8 at final follow-up [7]. Faure et al. reported a 31-case series with a median 13-year follow-up after a modular knee arthrodesis and found a mean VAS score of 3 [24]. Our results are in line with these similar studies in the literature, and support modular knee arthrodesis as a successful intervention in the relief of pain.

The OKS of this cohort showed a near 20-point gain with a mean final postoperative score of 33.8. Although this is not a perfect score of unhindered joint function, it represents a substantial improvement with only mild impairment relative to an excellent outcome. As patients experience less pain and mobilize better, this reflects on their OKS score. Since knee arthrodesis is considered a salvage method due to the loss of range of motion, some items in this questionnaire are presumably negatively affected. Gathen et al. evaluated a cohort of modular knee arthrodesis after failed revision TKA, and found a mean OKS score of 39.2 [6]. Friedrich et al. had a mean OKS score of 38 in their study of two stage arthrodesis of a modular intramedullary nail after septic TKA failure [5]. In another study, the mean OKS score of customized modular intramedullary nail arthrodesis cohort was found 41 ± 11, however, the series included patients with bony fusion and the preoperative values were not reported [14]. The results of our study appear relatively lower compared to these studies. As the minimal clinically important difference is 5 points in OKS, this should not be interpreted as a worse outcome [25, 26].

Patients with infected TKA may experience significantly lowered overall physical and mental well-being. The mean SF-12 Physical score of the cohort was 32.1, and the mean SF Mental score was found 40.2 at final assessment. Both scores showed improvements over the preoperative values, suggesting better outcomes. According to a similar study by Barton et al., knee arthrodesis had slightly higher SF-12 Physical scores compared to revision TKA (29.9 vs 28.4) [27]. The difference was even greater on SF-12 Mental scores, which were also in favor of knee arthrodesis (45.1 vs 36.5). Hungerer et al. reported similar SF-12 Physical scores between modular knee arthrodesis and above knee amputation (AKA) (30 vs 36), and proposed AKA as an alternative treatment [28]. Although AKA had slightly higher outcome scores in their study, they also led to a higher infection recurrence rate of 35%, compared to their 22% reinfection rate after arthrodesis. The study by Chen et al. reported that arthrodesis had better functional outcomes and ambulatory status compared to AKA, with SF-12 Physical scores of 51 against 26, respectively [29]. The results of our study were comparable to Barton and Hungerer et al.’s, which were slightly lower than the reports of Chen et al. These findings suggest that in terms of physical and mental well-being, the modular knee arthrodesis implant achieved comparable results to similar studies in the literature.

Avoidance or minimalization of leg length discrepancy (LLD) is a major advantage of the modular segmental arthrodesis system used in this study. LLD increases angular motion of the limbs, and LLD that exceeds 5.5% causes increased mechanical work [30]. The result of this is greater energy consumption during the gait cycle [31]. To compensate, patients lengthen the shorter limb by increasing pelvic obliquity, hip abduction, midstance knee extension, and walking in equinus; and shorten the longer limb by increased hip and knee flexion, ankle dorsiflexion, and circumduction [32]. LLD over 20 mm’s can also affect the spinal posture in the coronal plane [33]. To our knowledge, there are no studies that investigate the effect of LLD on gait after knee arthrodesis. We believe further studies in this area would be invaluable to the literature.

As an alternative to intramedullary techniques, arthrodesis with external fixators have been documented [8]. External fixators can also allow to lengthen the limb. Rozbruch et al. used external fixators for knee arthrodesis and simultaneous lengthening [34]. Their cohort had an average LLD of 1.8 cm at the end of treatment after a mean 5.4 cm lengthening. Instead of the acute lengthening provided by modular segmental systems, external fixators can only gradually counter the issue of LLD, and patients may have to stay frame-bound for a prolonged period. In addition, there are no means of bridging the bone defects, and bony fusion is necessary in the case of external fixators. In addition to the disadvantages of bone fusion (delayed weight bearing, LLD), external fixators have unique complications: pin-tract infections, and decreased mobility in elderly and comorbid patients [8]. In the same study by Rozbruch, the mean fixator duration was 11 months, and all of the patients experienced pin-tract infections [34].

The most common complication in our study was reinfection. A rate of infection recurrence after knee arthrodesis varies between 0 to 26% in the literature [5, 19]. In retrospective cohort study with 13 years of median follow-up, 26% of cases had undergone revision surgery due to reinfection [22]. They also reported that 75% of the recurrences occurred in the first 72 months. In the current study, one patient presented with septic implant failure, and there were no cases of aseptic loosening which were comparable to other similar studies in the literature. The modular shells can potentially create a surface for biofilm formation, the alternatives for the reconstruction of bone loss are limited. Massive allografts can fill the gap for a shorter or non-modular metal construct. Although theoretically this decreases the area that is prone to slime, allografts are not immune to the risk of infection [35]. Furthermore, a considerable number of multiply revised and chronically infected patients are malnourished, immunosuppressed, and have chronic comorbidities. Achieving union in such a population is often difficult, leading to delayed weight bearing status and functional recovery. Another complication encountered in this study was periprosthetic fracture. Although the use of stems allows stable fixation against angular moments and bypasses the deficient metaphyseal bone, they can also act as a stress riser and a long stem can lead to stress shielding in an already weakened bone, creating controversy about the ideal stem length. In spite of these, long stems have shown better results in clinical and biomechanical studies in the management of massive bone loss and unhealthy bone [36, 37].

There is no optimal method of managing such a catastrophic complication after total knee arthroplasty. The expectancy of inadequate functional recovery due to severe bone loss or soft tissue failures may preclude reimplantation of a revision TKA system. As such, knee arthrodesis can provide a feasible solution in a number of circumstances after failed infected TKA. In addition, revision TKA is an expensive procedure, and the exponential costs of repeating revision surgeries can strain overburdened healthcare systems [38]. However, as summarized previously, different options for arthrodesis have their own advantages and drawbacks. Surgeons should have detailed discussions with candidate patients regarding the rationale of the selected treatment method, and thoroughly inform them about the potential disadvantages and outcomes of the other options. Finally, long-term follow-up studies with a larger cohort is still needed to delineate the role of modular arthrodesis systems in the treatment of catastrophic infections after total knee arthroplasty.

This study has several limitations. The heterogeneity of the study group if difficult to control in such an uncommon procedure with rare indications. The multicenter retrospective nature of the study is another factor that hinders the execution of a completely standardized procedure. The number of patients in the cohort is low, thereby decreasing the power of the study. Further studies with larger patient populations will be necessary to make a more reliable comparison to the other treatment options.

## Conclusion

Uncontrolled infection after total knee arthroplasty can be effectively treated with arthrodesis using a modular intramedullary nail and satisfactory functional results can be obtained.

## Data Availability

The data that support the findings of this study are available on request from the corresponding author. The data are not publicly available due to containing information that could compromise the privacy of research participants.
